# Practical Inter-Floor Noise Sensing System with Localization and Classification

**DOI:** 10.3390/s19173633

**Published:** 2019-08-21

**Authors:** Junho Son, Chong-Min Kyung, Hyuntae Cho

**Affiliations:** 1 Samsung Electronics, Hwasung-Si 18448, Korea; 2 Department of Electrical Engineering, KAIST, Yuseong-Gu, Daejeon 34141, Korea; 3 Center for Integrated Smart Sensors, KAIST, Yuseong-Gu, Daejeon 34141, Korea

**Keywords:** acoustic noise sensor, inter-floor noise, microphone array, sound classification, sound pressure level, sound source localization

## Abstract

Inter-floor noise is a severe social problem which causes illegal arson, violence, and even murder. In this paper, an inter-floor noise sensing system is proposed to detect and record information related to inter-floor noise in an apartment building. The proposed system measured the noise level and estimated the direction of the noise source along with the type of noise. The noise level measurement is based on the sound pressure level (SPL) measurement, which is a logarithmic measure of the effective pressure of a sound relative to a reference sound pressure. Noise source localization was performed using the estimated time difference of arrival (TDOA) from the microphone array. For the classification of noise types, the Mel frequency cepstral coefficients (MFCC) and zero-crossing rate (ZCR) were extracted from a noise signal, and the k-nearest neighbor algorithm was used to classify the type of noise. In addition, we developed a noise monitoring hardware to evaluate our methods in the actual environment. The experimental results demonstrated that the proposed system had a reliable accuracy for each functional unit. The results showed that the error of the noise level was approximately ±1.5 dB(A), the error of the direction estimation was approximately ±10°, and the accuracy of the classification for the noise type was more than 75%. These output data from the proposed system are expected to be used as important reference data for any dispute cases due to inter-floor noise.

## 1. Introduction

Due to high population density, many countries have a very high rate of apartment housing. In the case of South Korea, the proportion of apartment house among all types of housing has continuously increased and the total percentage of apartment housing reached 75% in 2016 [1]. Roughly speaking, about three quarters of Korean citizens are sharing floors with their neighboring residents in their current housing situation. The unavoidable problem that arises from the high percentage of apartment housing is the inter-floor noise between neighboring residents.

Inter-floor noise in many countries has been a serious social problem. Conflicts between neighboring residents have occurred frequently, and some of them resulted in crimes such as arson, violence, and even murder. The governments of several countries created a legal standard related to inter-floor noise to provide a quantitative definition of it. The legal standard specifies different threshold levels of inter-floor noise according to their type and occurrence time [2,3].

A major problem of current situations is the lack of a specialized device for recording inter-floor noise even though there are a number of noise sensors in the market. With the currently available sound level meters, users can only measure the noise level without localization and classification [4,5,6,7]. In addition to the noise level, there are other more important data regarding inter-floor noise which can be used as reference data in dispute cases.

In this paper, an inter-floor noise sensing system, which detects not only the noise level, but also other meaningful information related to inter-floor noise is proposed. The proposed system captures and displays three pieces of information related to inter-floor noise: noise level, three-dimensional direction of the noise source, and type of noise. The noise level measurement is based on a logarithmic measure of the effective pressure of a sound relative to reference sound pressure, and the noise source localization is performed using the estimated time difference of arrival (TDOA) from the microphone array which consists of three pairs. For the classification of noise types, Mel frequency cepstral coefficients (MFCC) and zero-crossing rate (ZCR) were extracted from the noise signal, and k-nearest neighbor algorithm was used to classify the type of noise. Knowing the direction of the noise source helps in finding the location of the point where the inter-floor noise occurs frequently. The classification of the noise type is also important because the legal standard is different according to the type of inter-floor noise that is occurring.

The rest of this paper is organized as follows. Section 2 introduces related works. Section 3 illustrates how each function of the proposed system is implemented with detailed algorithms. Section 4 provides the experimental results of the proposed system. Finally, the conclusion of this paper is presented in Section 5 with recommendations for future studies.

## 2. Related Works

The proposed inter-floor noise sensing system contains three functional blocks: noise level, sour localization, and noise classification. In this chapter, we briefly introduce related works of each functional block of the proposed system.

### 2.1. Noise Level Measurement

Risojevic et al. [8] proposed accurate indoor sound level measurement on a low-power and low-cost wireless sensor node with limited computational resources. They implemented non-calibrated calculation of the sound pressure level and performed digital A-weighting filtering on the node. According to the experimental results, their approach could measure the noise levels of up to 100 dB with a mean difference of less than 2 dB compared to Class 1 sound level meters. Santini et al. [9] developed a system for the collection and logging of noise pollution data based on tiny sensor nodes. Two studies enabled real-time acquisition, processing, and visualization of data collected in wireless sensors networks. Zamora et al. [10] presented a Smartphone-based noise monitoring system. They focused their effort on the sound capture, processing procedure, and analyzing the impact of different noise calculation algorithms. They could measure the noise level of up to 95 dB and reduce average error below 2%. Most previous studies adopted the A-weighting function to improve the accuracy of the system [11,12]. It is defined as a standard in the international standard IEC 61672-1: 2003 [13]. They have complied with the requirement of the international standard in terms of accuracy. However, they did not consider noise source localization and the classification of noise types. These factors can be used as important reference data for any dispute cases due to inter-floor noise.

### 2.2. Sound Source Localization

The sound source localization technique is being widely used in various applications such as teleconferencing [14] and robot navigation [15,16]. There have been several investigations that attempted to localize the source of the sound. Among those approaches, the sound source localization based on time difference of arrival (TDOA) from microphone arrays [17,18,19,20,21] is the most widely used and reliable technique. In the proposed sound localization system, the TDOA estimated from the microphone array was used to localize the direction of the noise source. The microphone array structure consisted of several pairs of microphones. Basically, one microphone pair was an angle sensing sensor of the incoming sound signal. To estimate the incident angle of sound, it was assumed that the distance to the sound source is much larger than the interval of the microphones such that incoming sound waves can be considered as coming in parallel [22,23,24].

Other studies [25,26] presented localization systems based on the time difference of arrival on Zigbee networks. They achieved high accuracy within several meters even though they use 2.4 GHz radio frequency. These approaches require accurate time synchronization since their localization methods are based on multiple sensor nodes.

### 2.3. Sound Classification

**Figure 1:**
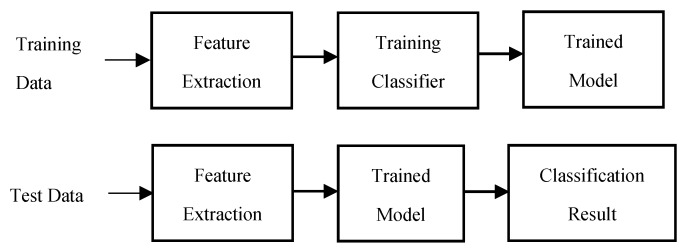
Basic methodology of classification task.

The sound classification technique is also widely used in various fields of applications such as environmental sound recognition [27,28] and speech recognition [29]. Figure 1 provides the basic steps of the classification task. In order to perform the classification, the first requirement is to train a classifier. Meaningful information is extracted from the training data, and this meaningful information is often referred to as a feature. Features are extracted from the raw data because the raw data contain a considerable amount of redundant information and reduction in the dimensionality of the data for later computation is desired. The extracted features were placed onto the feature space, and ‘clustering’ of the labeled data was conducted using the extracted features. This process represents the training of a classifier. The trained model was created as a result of the training process, and the test data was classified in a trained model.

After the feature extraction, a classifier should be selected to train a model from the extracted features. There are several classifiers widely used in the field of machine learning such as k-nearest neighbor (k-NN), support vector machine (SVM), convolutional neural network (CNN), recurrent neural network (RNN), and artificial neural network [30,31,32,33,34].

## 3. Implementation of the Inter-Floor Noise Sensing System

**Figure 2:**
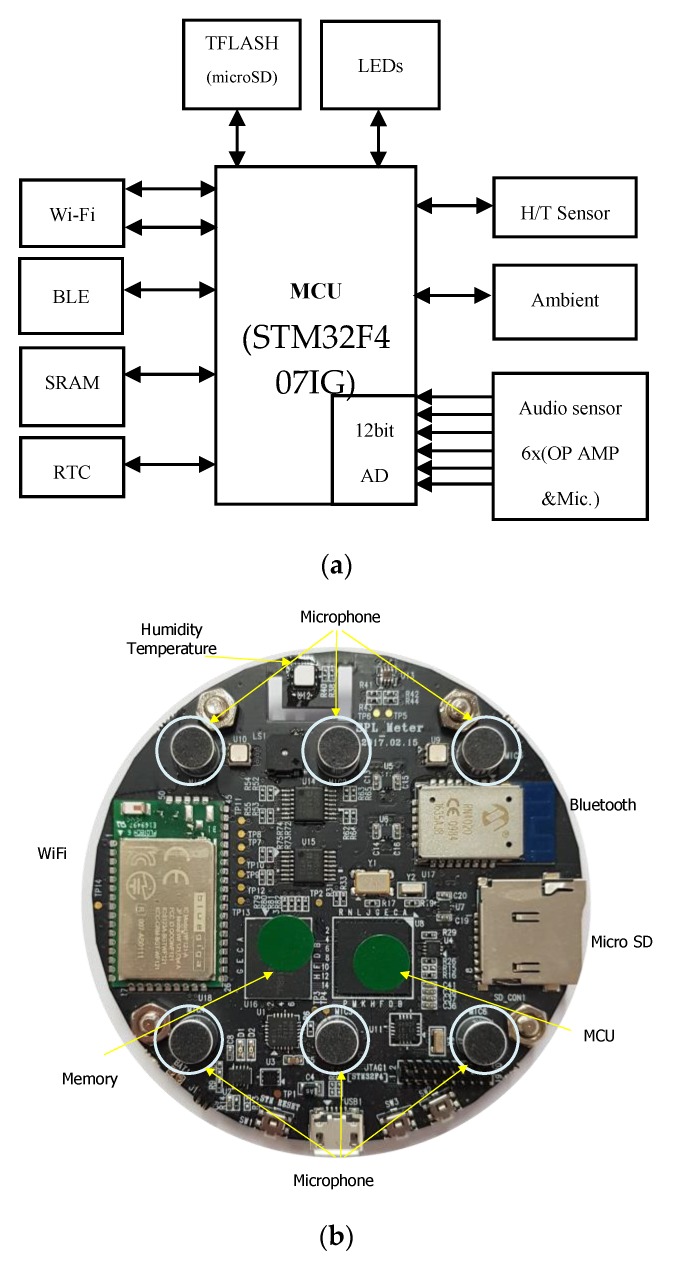
Sensor board of the system: (**a**) block diagram and (**b**) hardware prototype.

First, a hardware system was designed and implemented to measure the noise by using a microphone array. Figure 2 shows the block diagram and appearance of the system. The system uses a STM32F407IG MCU based on an ARM 32-bit Cortex-M4 MCU that operates at 120 MHz. A 12 bit analog-to-digital converter (ADC) is used to convert analog the audio signal into digital data. Additionally, it contains 2 MB of extra SRAM to improve the computing power and has an external flash memory to store the noise data over the long term. Other components, such as the humidity, temperature sensor, and Bluetooth module are used for our convenience. The inter-floor noise measurement function and sound localization function are implemented in the designed hardware system. The output information (i.e., noise level and direction of the noise) with the raw data is transferred to PC and displayed in real time.

### 3.1. Measurement of Noise Level

**Figure 3:**
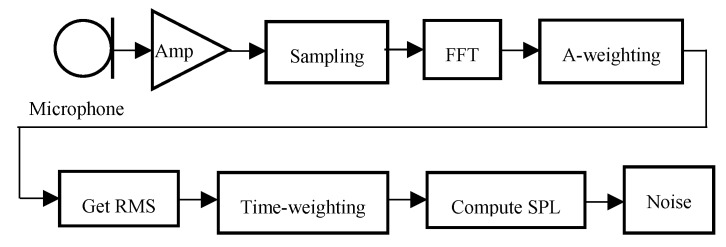
Flow chart of the sound pressure level (SPL) measurement unit.

The procedure for estimating the noise level from the noise signal is shown in Figure 3. First, an analog audio signal is converted into an analog voltage signal through a microphone. Sensitivity of the microphone is −45 dB and operating voltage is 2 V. The analog voltage signal is sampled and converted into digital data through the ADC. The fast Fourier transform (FFT) is performed on the digitized samples. In the frequency domain, the A-weighting is applied to consider the human’s different perceptions of different frequency levels. After that, the root mean square (RMS) of the signal is calculated. The time weighting is applied to smoothen the displayed data, and finally the sound pressure level (SPL) of the noise is calculated. A detailed explanation of each step will be presented in the following subsubsections.

#### 3.1.1. Amplifying Circuit

**Figure 4:**
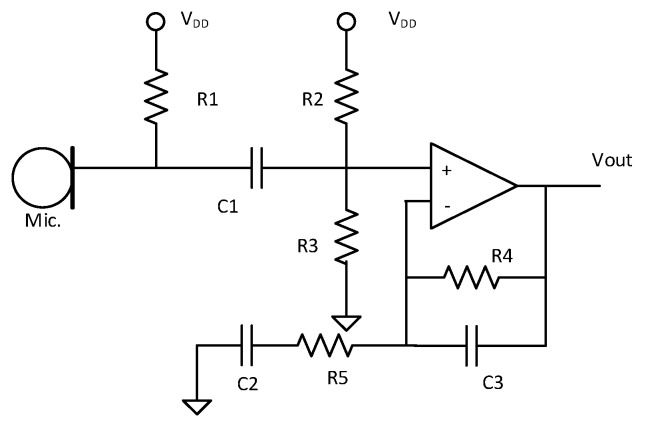
Amplifying circuit for the microphone.

Since the output voltage level of the microphone is very low, an amplifying circuit is needed to amplify the voltage produced from the microphones. In the designing process, two things needed to be considered: gain and bandwidth. Gain and bandwidth can be adjusted by changing the values of the resistor and capacitor in the feedback circuit. The audio amplifying circuit in the sensor board is shown in Figure 4. The gain of the amplifying circuit is 20 dB, and the 3 dB cut-off frequency is approximately 11 kHz. The bandwidth of the amplifying circuit should exceed at least 8 kHz, which is a typical frequency range of commercial SPL meters. Therefore, the feedback gain and cut-off frequency should be considered according to the target noise level and bandwidth.

#### 3.1.2. Sampling and FFT 

An amplified voltage signal is sampled in a 12 bit-ADC of the sensor board. The sampling rate is 200 kHz in the proposed system. A much higher sampling rate is used compared to the conventional audio sampling rate (44.1 kHz). This approach was chosen because later in the localization system, a high enough sampling frequency is required to compensate for the very short distance between the microphones (5.7 cm). The Cooley–Tukey algorithm [35], specifically radix-2 FFT is used to calculate the FFT.

#### 3.1.3. A-Weighting

A-weighting is applied to consider the human ear’s different sensitivity of the different frequencies of sound. The international standard [13] defines the frequency weightings by tables for certain frequencies; but there are appropriate functional realizations of the frequency weightings:

(1) $$R_{A}\left(f\right)=\frac{12194^{2}\cdot f^{4}}{\left(f^{2}+20.6^{2}\right)\sqrt{\left(f^{2}+107.7^{2}\right)\left(f^{2}+737.9^{2}\right)}\;\left(f^{2}+12194^{2}\right)},$$

(2) $$A\left(f\right)=20\;\log_{10}\left(R_{A}\left(f\right)\right)+2.0.$$

We apply the A-weighting in the frequency domain by calculating the appropriate values of *A*(*f*) for each frequency component.

#### 3.1.4. Time-Weighting 

After the A-weighting, the RMS voltage of the A-weighted signal is calculated. Then, a time-weighting is applied on the time series of the RMS voltage. A time-weighting is needed to smoothen the output displayed data. The displayed data cannot be read if the rate of data change is too fast. The time weighting is sometimes called exponential averaging, and it acts as low-pass filters to remove the high frequency noise. In the proposed system, the time-weighting of the RMS voltage is expressed as the following equation:

(3) $$V_{RMS}=\alpha V_{RMS\_new}+\left(1-\alpha \right)V_{RMS\_old},$$

where α in the above equation is the smoothing factor, and 0 < α < 1. The lower values of α indicates smoother data displays. The value of α is given by:

(4) $$\alpha =1-\exp\left(\frac{-\Delta T}{\tau }\right),$$

where *ΔT* is the sampling rate of the data and τ is time as a constant. Two types of time constants are often used to display the sound pressure level: F (Fast, 125 ms) and S (Slow, 1 s). Both time constants are implemented in the proposed SPL measurement unit.

#### 3.1.5. Computing SPL

The final step is to compute the sound pressure level of the signal. It is calculated as:

(5) $$\mathrm{SPL}=20\;\log_{10}\frac{V_{RMS}}{V_{REF}}\;\left[\mathrm{dB}\left(A\right)\right],$$

where $V_{REF}$ is the output RMS voltage when the pressure level of the sound signal is equal to the reference sound pressure $P_{0}$ (20 micro-pascal). $V_{REF}$ is determined from the microphone sensitivity and gain in the amplifying circuit. 

### 3.2. Localization of Noise Source 

#### 3.2.1. Introduction to Time Difference of Arrival 

**Figure 5:**
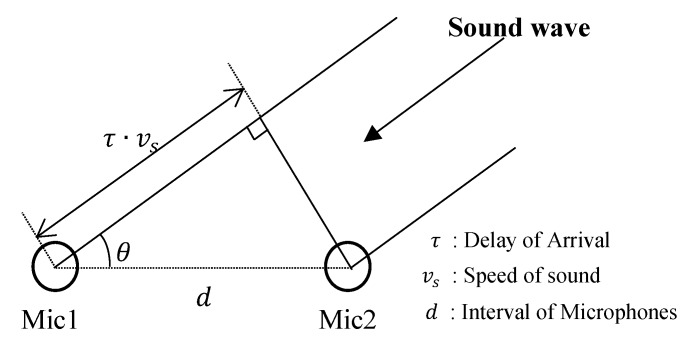
Basic geometry of a microphone pair.

The basic geometry of a microphone pair is shown in Figure 5. Basically, one microphone pair was an angle sensing sensor of the incoming sound signal. To estimate the incident angle of sound, it was assumed that the distance to the sound source is much larger than the interval of the microphones such that incoming sound waves can be considered as coming in parallel, This assumption is reasonable because the interval of the microphones of our system was only 5.7 cm; while, the typical distance to the inter-floor noise source will be at least more than 10 times of the interval of the microphones. The direction angle, *θ*, to the sound source in Figure 5 can be calculated by the following equation:

(6) $$\theta =\cos^{-1}\left(\frac{\tau_{delay}\cdot v_{s}}{d_{mics}}\right).$$

The TDOA of a microphone pair can be derived from the cross-correlation matrix of samples acquired from each microphone. Generally, the cross-correlation is a measure of similarity of two signals as a function of the displacement of one relative to the other. In the time series analysis, a cross-correlation is often used for estimating the time delay between two signals [24]. The cross-correlation of two sound signals acquired from each microphone *i* and *j* is expressed as:

(7) $$Corr_{i,j}\left[\tau \right]=\overset{N-1}{\underset{n=0}{\sum }}x_{i}\left[n\right]x_{j}\left[n-\tau \right],$$

where $x_{i}\left[n\right]$ is the signal captured by microphone *i*. In digital signal processing, the cross-correlation is often calculated in the frequency domain. The major advantage of calculating the cross-correlation in the frequency domain is the reduction in the computation cost due to the use of a fast Fourier transform (FFT). In the frequency domain, cross-correlation is calculated as:

(8) $$Corr_{i,j}\left[\tau \right]=FT^{-1}\left[FT^{*}\left[x_{i}]\cdot FT[x_{j}\right]\right],$$

where FT[ ] and $FT^{-1}\left[\;\right]$ is the Fourier Transform and the inverse Fourier Transform, respectively. $FT^{*}\left[\;\right]$ denotes the complex conjugate of the Fourier Transform. The maximum of the cross-correlation function indicates the point where the signals are best aligned, and the argument of the maximum refers to the delay between the two signals. The delay of arrival between the two signals from microphone *i* and *j* is derived as:

(9) $$\tau_{delay}=\arg\underset{t}{\max}\left(Corr_{i,j}\left[t\right]\right).$$

We should note that the above result is the delay in a sample if the cross-correlation is computed in a sample domain. To convert the delay in a sample to delay in time, the division of the delay in sample by sampling frequency of the system is required.

#### 3.2.2. Microphone Array Structure 

**Figure 6:**
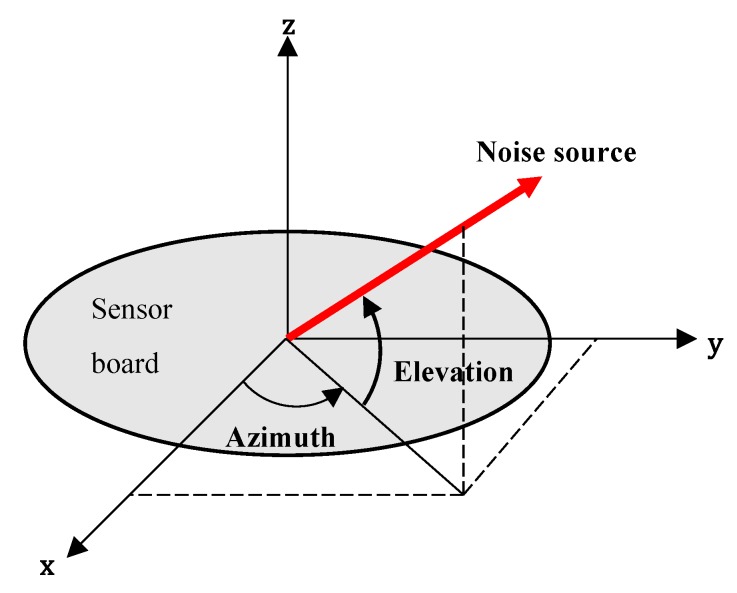
Two output angles of the localization system: Azimuth and elevation.

The localization system is roughly divided into two parts. The first part is obtaining the TDOA from the microphone pairs, and the second part is identifying the direction angles from the estimated TDOA. In our localization system, a three-dimensional direction of the sound source is expressed as two angular components: azimuth and elevation. Figure 6 illustrates the two output angles in local Cartesian coordinate of our system.

**Figure 7:**
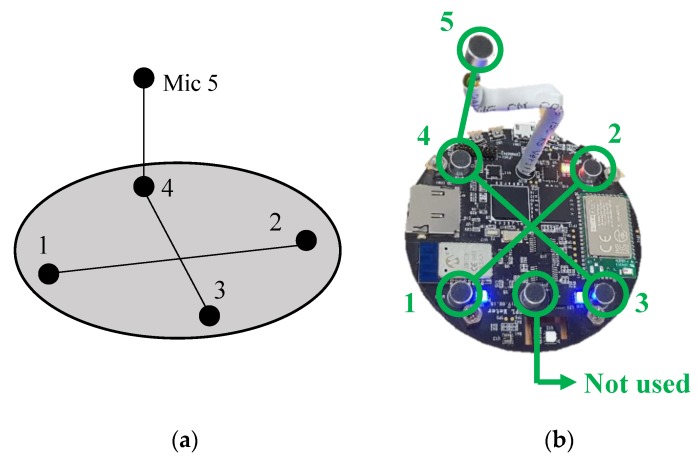
Pairing of the microphones on the sensor board: (**a**) simplified microphone array structure and (**b**) actual hardware system.

There is a total of six microphones on the hardware system, and only five are used in the proposed microphone array structure. Figure 7 depicts the microphone array structure of our system. A total of five microphones on the sensor board are used to identify the noise source direction in a three-dimensional space. In Figure 7b, the microphones that are connected by a common line formed one microphone pair. Three microphone pairs are used to estimate the TDOAs of the input noise signal. Microphone pair 1 (Mic 1 and 2) and 2 (Mic 3 and 4) are used to estimate the azimuth and elevation. Microphone pair 3 (Mic 4 and 5) is used to remove ambiguity of the *z*-axis (decision of *z* > 0 or *z* < 0) when the elevation is estimated.

#### 3.2.3. Estimation of Azimuth and Elevation

**Figure 8:**
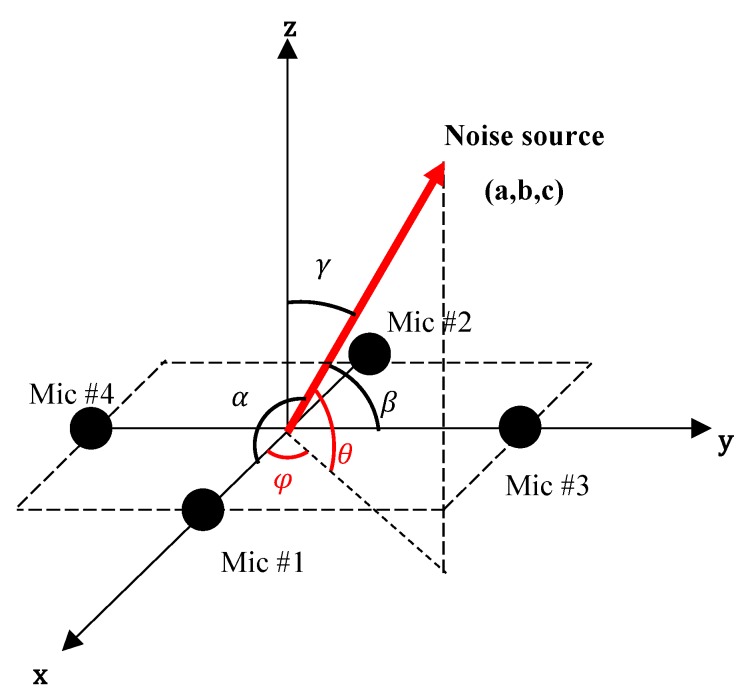
Direction angles in the local Cartesian coordinate.

The angles of the noise source direction vector with respect to the *x*, *y*, and *z* axes in a local Cartesian coordinate is exhibited in Figure 8. The α, β, and γ are the direction angles of the noise source vector regarding the *x*, *y*, and *z* axes, respectively. The φ and θ denote the azimuth and elevation, respectively. Our goal is to identify the azimuth φ and elevation θ from the direction angles α and β.

From the TDOA estimated from each microphone pair, the value of α and β are calculated by:

(10) $$\alpha =\cos^{-1}\left(\frac{TDOA_{mic1,2}\cdot v_{s}}{d_{mics}}\right),$$

(11) $$\alpha =\cos^{-1}\left(\frac{TDOA_{mic1,2}\cdot v_{s}}{d_{mics}}\right).$$

Following the relationship between the direction cosines of the noise direction vector holds:

(12) $$\cos^{2}\alpha +\cos^{2}\beta +\cos^{2}\gamma =1.$$

The relationship between the elevation θ and *z*-axis direction angle γ is:

(13) $$\theta =90^{\circ }-\gamma .$$

We can derive an expression for the elevation from above the two equations:

(14) $$\theta =90^{\circ }-\cos^{-1}\left(\sqrt{1-\cos^{2}\alpha -\cos^{2}\beta }\right).$$

For azimuth, the tangent of azimuth is expressed as:

(15) $$\tan\varphi =\frac{b}{a}=\frac{\cos\beta }{\cos\alpha }\;.$$

We can derive an expression for azimuth as:

(16) $$\varphi =\tan^{-1}(\frac{\cos\beta }{\cos\alpha }).$$

If only two microphone pairs are used to identify the elevation, the ambiguity between the *z* > 0 plane and *z* < 0 plane exits. To enable a full 3D space localization, one additional microphone pair along the *z*-axis is used. The sign of the TDOA of microphone pair 3 determines where the noise source is located, either the *z* > 0 space or *z* < 0 space. Considering this issue, the expression of elevation is modified as:

(17) $$\theta =sgn\left(TDOA_{mic4,5}\right)\left[90^{\circ }-\cos^{-1}\left(\sqrt{1-\cos^{2}\alpha -\cos^{2}\beta }\right)\right].$$

### 3.3. Classification of Noise Types 

The procedure for classifying the noise type is basically the same as the procedure explained in Section 2. The first requirement is to extract the meaningful audio features from the audio data. This part is essential because the raw audio data contains a great deal of redundant information. There are several features used in sound classification or recognition. Among those, the most popular and widely used audio feature is the Mel Frequency Cepstral Coefficients (MFCC).

The MFCCs refer the coefficients of the MFC (Mel-frequency cepstrum), and the MFC is a representation of the short-term power spectrum of a sound on the Mel scale. The Mel scale, whose name comes from the word melody, is a perceptual scale of pitches judged by listeners to be equal in distance from one another. As a human’s perceptions of pitch intervals are more sensitive in the low frequency region, the Mel scale is a non-linear function in frequency representation. A formula to convert *f* (Hz) into the *m* (Mel scale) is:

(18) $$m=2595\;\log_{10}\left(1+\frac{f}{700}\right)=1127\ln\left(1+\frac{f}{700}\right).$$

**Figure 9:**
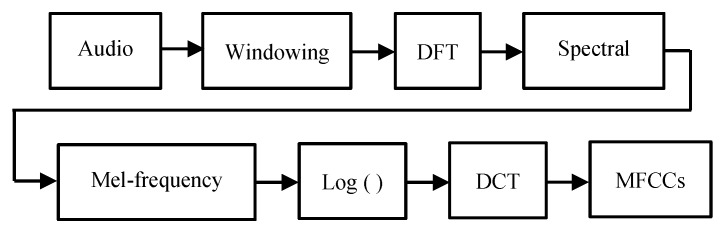
Flow chart of extracting Mel Frequency Cepstral Coefficients (MFCCs).

Figure 9 displays the flow chart for extracting the MFCCs from audio data. Prior to the MFCC extraction, an audio clip was divided into short-time frames. The typical frame length was usually from 10 to 30 ms with a 50% overlap between the adjacent frames.

After framing, a window function was applied to each frame. A popular choice for a window function is the Hamming window. The Hamming window is defined as:

(19) $$w\left[n\right]=0.54-0.46\cos\left(\frac{2\pi n}{N-1}\right),$$

where *N* is the window length. After windowing, a FFT was conducted on each frame. The next step was to apply the triangular filters onto the Mel-scale. Each filter in the filter bank had a triangular form and an amplitude of 1 at the center frequency. The intervals between the center frequencies of each filter was set to be equal in the Mel-scale. The final step was to do a discrete cosine transform (DCT) after taking the logarithm of the filtered spectral magnitudes. As a result, the MFCCs from each filter were obtained. Only the first 12~13 MFCCs were chosen because the higher indexed MFCCs usually did not contain useful information for sound classification.

#### 3.3.1. Training Dataset

For training purposes, the public audio dataset named ESC-50 was used [36]. ESC-50 contains 2000 annotated audio clips for environmental sound classification. The dataset is composed of 50 classes of various environmental sounds and each class consists of 40 audio clips. Each audio clip is 5 s long and the sampling rate is 44.1 kHz.

Among the 50 classes, seven classes were chosen that are common types of inter-floor noise in an apartment house. Those classes are (1) footsteps, (2) door knock, (3) dog barking, (4) baby crying, (5) clock alarm, (6) vacuum cleaner, and (7) washing machine. According to the legal standard in Table 1, footsteps and door knock are noises occurring from direct impact, while the rest of the classes are classified as noise delivered through air.

#### 3.3.2. Training Process

**Figure 10:**
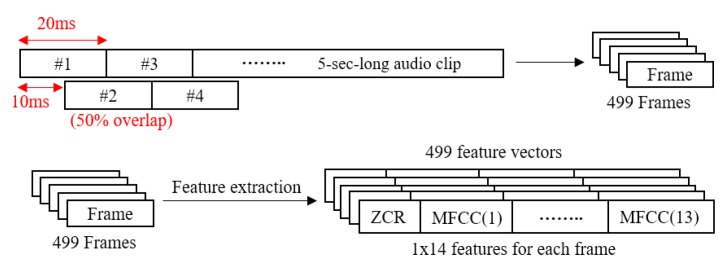
Feature extraction from one audio clip.

The feature extraction process of one audio clip is illustrated in Figure 10. First, the original audio clip was divided into 20 ms-long sub-frames with 50% overlap. A total of 499 short frames were obtained from one 5 s-long audio clip.

Generally, the MFCCs feature is a typically used audio classification. However direct impact, such as hammering and children leaping, needed to be detected. Therefore, a combination of the MFCCs and zero-crossing rate is used in our classification system. The zero-crossing rate is the rate of sign-changes along a time signal as its name states. It is known to be a powerful feature in classifying percussive sounds [37]. Just like the MFCCs, audio data is divided into short-time frames prior to feature extraction. For each divided frame, the zero-crossing rate is computed. The zero-crossing rate of the digital audio signal *x*[*t*] is calculated as following equation:

(20) $$\mathrm{ZCR}\left(x\left[t\right]\right)=\frac{1}{2\left(N-1\right)}\overset{N-1}{\underset{i=1}{\sum }}\left\{sgn\left(x\left[i\right]\right)-sgn\left(x\left[i-1\right]\right)\right\}$$

These two features are extracted from each frame. For MFCCs, 13 MFCCs were chosen and the remainder were discarded. 

As a result, the 499 × 14 feature table was obtained from one audio clip (size of 1 × 220,500). This result is a 97% reduction of the data size. Since the zero-crossing rate and MFCCs are not on the same scale, they needed to be normalized, which was conducted by subtracting the mean and dividing by the standard deviation of each column of the feature table. After the feature extraction, a classifier was trained with the obtained features. A k-NN classifier [38] with *k* = 5 was used in the proposed system. As its name states, the k-NN algorithm identifies the k nearest neighbor data from the input data in the feature space. The output class was determined as the most common class type among its k nearest neighbor data.

## 4. Experimental Results

### 4.1. Measurement of Noise Level

**Figure 11:**
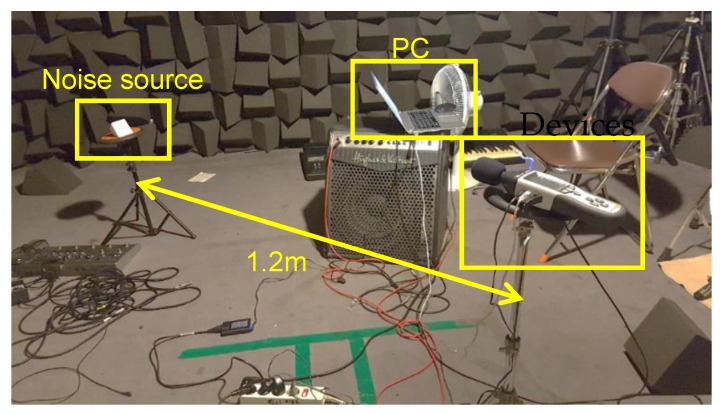
Experimental setup for SPL measurement.

To evaluate the accuracy of the noise level, the SPL value obtained from the proposed system was compared with a commercial SPL meter (DT-8852) [39] as a reference. Figure 11 shows an experimental environment for performance evaluation. The DT-8852 has an error of ±1.4 dB(A). Experiments were performed in an anechoic chamber. A Bluetooth speaker was used to generate sound signals, and the distance between the speaker and noise monitoring devices was set to 1.2 m. Pure-tone sinusoidal sound waves with frequencies that ranged from 500 to 8000 Hz were used since the frequency range of commercial SPL meters is usually up to 8000 Hz. The experimental environment in the anechoic chamber and reference SPL meter were used in the experiment.

**Figure 12:**
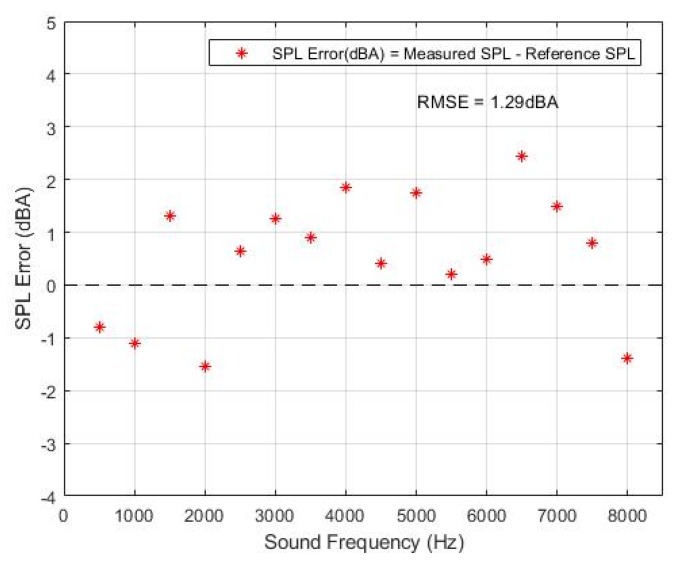
Error of the SPL measurement for different frequency levels.

The measurement error was calculated by subtracting the reference SPL from measured SPL. A total of 16 sound signals with different frequencies were used in the experiment. The frequency intervals of each sound signal was 500 Hz. Figure 12 shows the measurement error of the proposed system compared to the reference SPL meter. The maximum deviation from the reference value did not exceed 2 dB(A) with the exception of the signal at 6500 Hz. To illustrate the system accuracy in one value, the RMS error from each result was computed. The resulting RMS error in the experiment is 1.29 dB(A).

### 4.2. Localization of Noise Source

The accuracy of the estimated azimuth and elevation from the proposed system was evaluated in the experiment. The experiment was performed in a reverberant room to provide more realistic conditions of an apartment building environment. A 1 kHz pure-tone sound wave was used as a noise signal. The distance between the speaker and our system in the experiment was set to 50 cm.

**Figure 13:**
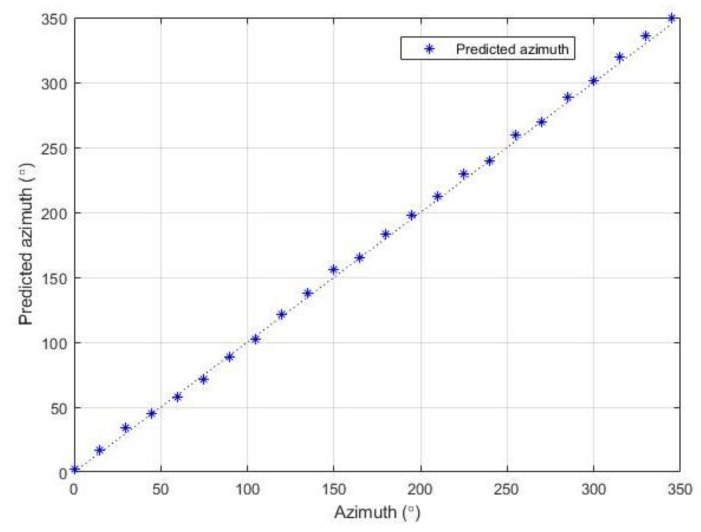
Predicted azimuth values compared to actual values.

In the experiment regarding the estimation of azimuth, the noise source (speaker) was placed at each azimuth from 0° to 345°. Each test angle was 15° apart from each other, so the experiment was performed for 14 different azimuth values. The elevation was set to 0° in the experiment. Figure 13 shows the result of the predicted versus actual azimuth values. The dashed blue line is the ground truth value and blue dots indicate the estimated azimuth values from the system.

**Figure 14:**
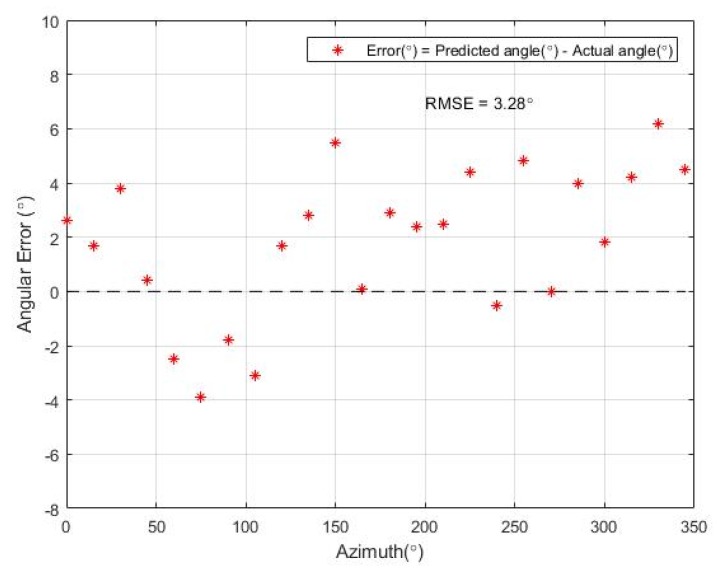
Estimation errors of azimuth for different positions.

Similar to the SPL error in the previous experiment, the measurement error of the azimuth prediction is calculated as the difference between the predicted and actual azimuth values. Figure 14 illustrates the distribution of the estimation error at each azimuth value.

The most deviations occurred in the ±5° range from the ground truth. The RMS error of the predicted azimuth value was computed, and the resulting RMS error in the experiment was 3.28°.

**Figure 15:**
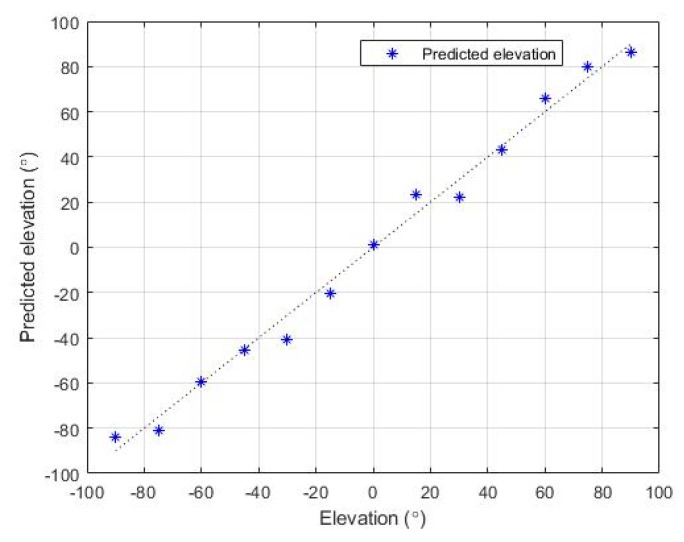
Predicted elevation values compared to actual values.

**Figure 16:**
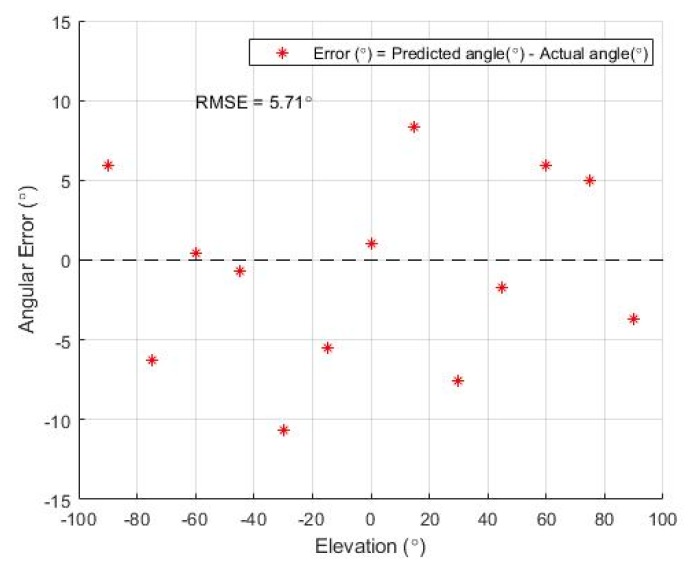
Estimation errors of elevation for different positions.

In the experiment regarding the estimation of elevation, the noise source (speaker) was placed at each elevation value from −90° to 90°. Each test angle was 15° apart from each other. Figure 15 shows the predicted azimuth values for each of the actual azimuth values. The dashed line indicates the ground truth and the blue dot is the predicted elevation for each position. Figure 16 illustrates the distribution of the estimation error at each elevation position. The accuracy of the estimated elevation is lower compared to the azimuth estimation. This outcome is due to the system not using the information from the microphone pair along the *z*-axis in the estimation process. The angular error is calculated as the difference of the predicted and actual angles. The resulting RMS error of the elevation estimation is 5.71°.

### 4.3. Classification of Noise Type

We used seven classes of the ESC-50 dataset for the noise type classification. As earlier mentioned, the selected seven classes were a clock alarm, crying baby, dog barking, door knock, footsteps, washing machine, and vacuum cleaner. They undergo the proposed hardware system to extract exact features. Audio clips in the ESC-50 dataset were used as the training set in our classification system. Two kinds of features (MFCCs and zero-crossing rate) were extracted from each audio clip, of which formed a feature space. The type of inter-floor noise of the test audio clip was determined using k-NN classifier (*k* = 5).

**Figure 17:**
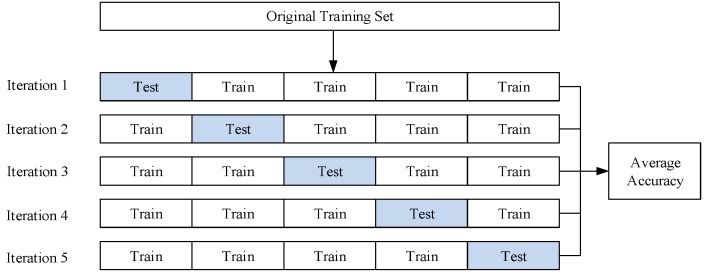
Diagram of five-fold cross-validation.

The accuracy of our trained model was evaluated first by performing a five-fold cross-validation. The accuracy obtained by the cross-validation indicates how accurately a trained model performs in test situations. The conceptual diagram of a five-fold cross-validation is shown in Figure 17. In a five-fold cross-validation, the training set is randomly divided into five subsets. Of five subsets, one is used as a test set, while the other subsets are used as training sets. Iteration is repeated until all of the subsets are used once as a test set. Finally, cross-validation accuracy is obtained by averaging the accuracies from all of the iteration processes.

**Figure 18:**
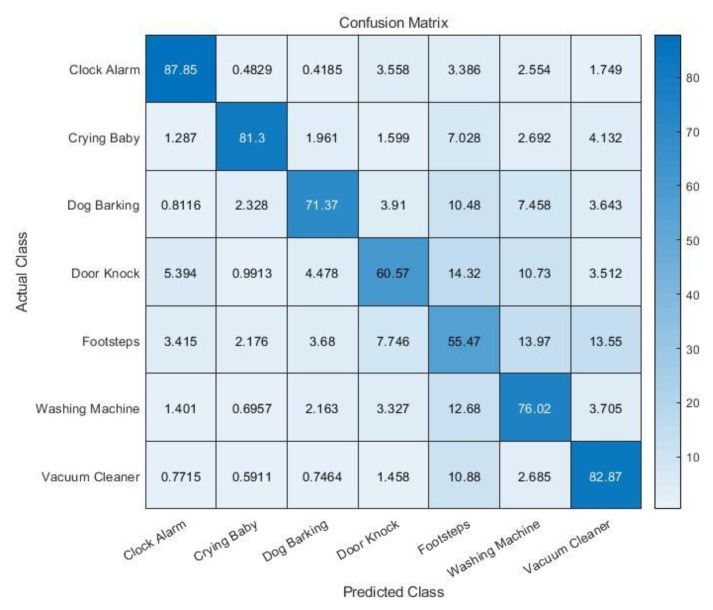
Five-fold cross-validation accuracy (MFCC only).

The classifier was trained in two different ways to see the effect of the zero-crossing rate as a training feature. First the model was trained by using only MFCCs; then a second model was trained by using both the MFCCs and zero-crossing rate. Figure 18 displays the cross-validation accuracy of the trained model (MFCC only). The average cross-validation accuracy of the trained model is 73.6%.

**Figure 19:**
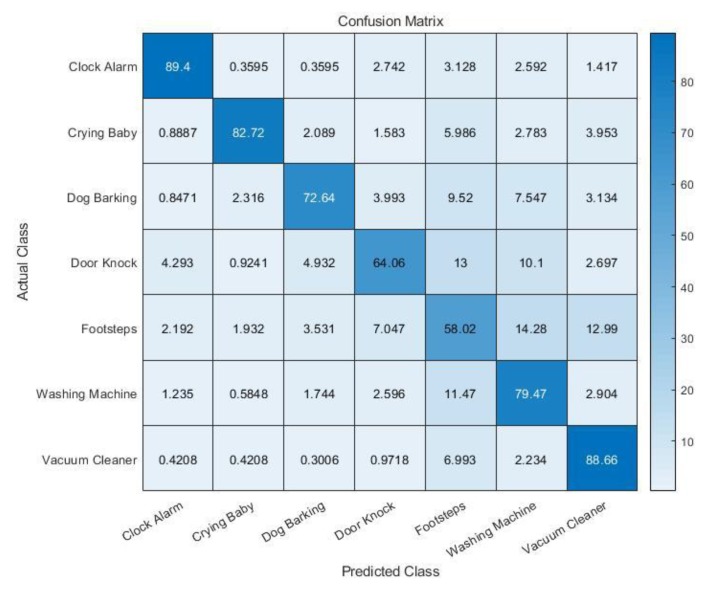
Five-fold cross-validation accuracy (MFCC + zero-crossing rate).

Figure 19 shows the cross-validation accuracy of the second model (MFCC + zero-crossing rate). Among the seven classes, the sounds from direct impact, such as a door knock and footsteps, had relatively low accuracy. The average accuracy of the five-fold cross-validation is 77.3%, which is about 4% higher than the accuracy of the first model. This result demonstrates that the use of the zero-crossing rate improves the accuracy of the classifier.

**Figure 20:**
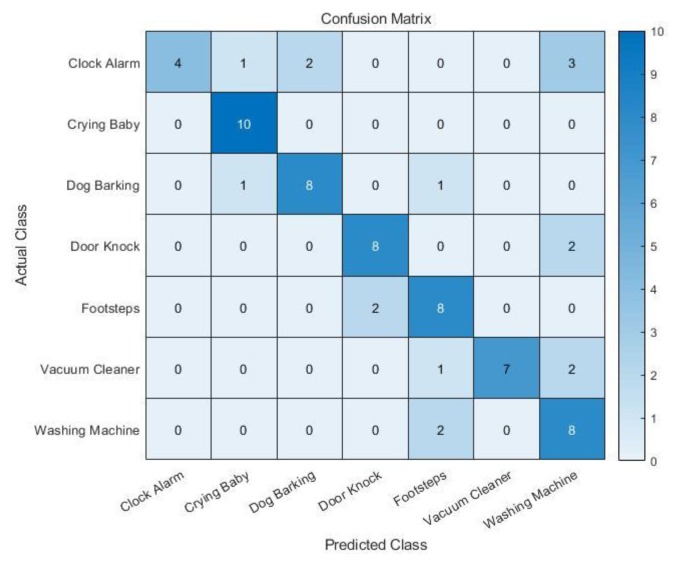
Test results of the trained system.

A test was conducted after training a classifier. As a test set, the sound samples of the seven classes were primarily collected from YouTube. Then, the actual output sound underwent our hardware system to contain hardware dependent features. For the clock alarm sound, due to the lack of appropriate samples on YouTube, some were manually recorded. The collected sound clips were resized into 5 s long clips using a software named Audacity [40]. As a result, 10 audio clips were prepared for each class as a test set. Figure 20 shows the classification results of the test set. The numbers inside the confusion matrix simply indicates the number of audio clips which belonged to a specific cell of the confusion matrix.

Among the 10 samples, more than seven samples are classified correctly, with the exception of the clock alarm sound. The classification accuracy of the clock alarm sound is relatively low. This result might be from the lack of variety among the training data for clock alarms. Most of the clock alarm training data just contained a ‘beep’ sound; while the test data of clock alarms contained various kinds of clock alarm signals. The accuracy in the classification indicates the rate of true predictions among all of the samples. The accuracy of each class is given by:

(21) $$\mathrm{Accuracy}\;\left(\%\right)=\frac{\mathrm{Number}\;\mathrm{of}\;\mathrm{correctly}\;\mathrm{predicted}\;\mathrm{samples}}{\mathrm{Total}\;\mathrm{number}\;\mathrm{of}\;\mathrm{samples}}.$$

Table 1 shows the classification accuracy of each class on a test set. Except clock alarm, the classes have acceptable accuracy. The average classification accuracy on a test set is 75.7%, which is similar to a five-fold cross-validation accuracy.

A limitation of the current classification system is the lack of training data. Our target classification classes are inter-floor noises; while the training data used in this experiment is for general environmental sound classification. For example, in the footsteps class, the training data contained not only indoor footsteps, but also footsteps in an outdoor environment, such as a gravelly field. Therefore, the dataset is not optimized for the target purpose of our system. The accuracy of the system can improve further if the training dataset from real inter-floor noise is used.

## 5. Conclusions

In this paper, an inter-floor noise sensing system which captured and displayed important information regarding inter-floor noise was proposed. The proposed system measured the noise level and estimated the direction of the noise source and type of noise. An embedded system with a microphone array was used to capture the noise signal. The noise level measurement and localization system were implemented on the sensor board. The classification system was implemented in a PC due to the lack of computing memory of the sensor board.

Experiments were performed for each functional block to evaluate the accuracy of the proposed system. The SPL measurement unit of the system was demonstrated to have a SPL RMS error of 1.29 dB(A). In the localization system, azimuth and elevation were estimated. The accuracy was evaluated for both angles, and the results showed RMS errors of 3.3° and 5.7° for azimuth and elevation, respectively. In the classification system, a public audio dataset was used as a training set. A five-fold cross-validation of the trained model demonstrated an accuracy of 77.3%, and the test results of the manually collected sound samples had an accuracy of 75.7%. To conclude, the proposed noise sensing system had reliable accuracy for all three functional blocks. The detected information from the proposed system are expected to be used as important reference data for any dispute cases due to inter-floor noise.

Future studies should include further calibration with humidity, temperature, and altitude. We should also improve classification performance by adopting other methods such as RNN, CNN, or deep learning. 

## Figures and Tables

**Table 1:** Classification accuracy of the test set.

Class	Accuracy (%)
Clock alarm	40
Crying baby	100
Dog barking	80
Door knock	80
Footsteps	80
Vacuum cleaner	70
Washing machine	80
Average	75.7
